# The complete mitochondrial genome of the tea lace bug, *Stephanitis chinensis* (Hemiptera: Tingidae)

**DOI:** 10.1080/23802359.2017.1372716

**Published:** 2017-09-06

**Authors:** Pin-Wu Li, Xiao-Qing Wang, Shi-Chun Chen, Ping Peng

**Affiliations:** a College of Horticulture, Sichuan Agricultural University, Sichuan, P. R. China;; b Tea Research Institute of Chongqing Academy of Agricultural Science, Chongqing, P. R. China

**Keywords:** Mitochondrial genome, *Stephanitis chinensis*, Tingidae, tea pest

## Abstract

The tea lace bug, *Stephanitis chinensis* Drake (Hemiptera: Tingidae), is a pest which feeds on the undersides of tea leaves by piercing the epidermis and sucking the sap, and causes great harm to plant growth and tea production. We have obtained the whole mitochondrial genome of *S. chinensis* (GenBank accession No. MF498769). The entire mt genome is 16,667 bp in size with an A + T content of 78.41%. The tea lace bug mt genome encodes all 37 genes that are typically found in animal mt genomes, consists of 13 protein-coding genes (PCGs), 2 ribosomal RNA, and 22 transfer RNA genes. The gene order is consistent with other sequenced mt genome of lace bugs. The A + T-rich region of this genome is 2215 bp long with the A + T content of 82.58%, and located between the *rrnS* and *trnI* genes. Phylogenetic analysis performed using 13 PCGs with 14 heteropteran insects showed that *S. chinensis* clusters with other Tingidae species.

The tea lace bug, *Stephanitis chinensis* Drake (Hemiptera: Tingidae), is a pest which feeds on the undersides of tea leaves by piercing the epidermis and sucking the sap, and causes great harm to plant growth and tea production. In this study, adults of *S. chinensis* were collected from a tea plantation of Chongqing province of China in May 2016, and stored in the insect specimen room of Tea Research Institute of Chongqing Academy of Agricultural Science with an accession number CQNKY-HE-01-01-01.

The complete mitochondrial genome of *S. chinensis* is a typical closed-circular DNA molecule with a full length of 16,667 bp, and the annotated sequence file was submitted to NCBI (GenBank accession No. MF498769). This genome is the largest mt genome among all sequenced Tingidae species: *Corythucha ciliata* (15,257 bp) (Yang et al. 2013) and *Pseudacysta perseae* (15,850 bp). The total nucleotide composition of the major strand of the mt genome is as follows: A = 43.88% (7314), C = 11.95% (1991), G = 9.65% (1608), and T = 34.52% (5754), with a total A + T content of 78.41% that is heavily biased toward A and T nucleotides. AT-skew and GC-skew of the whole J-strand of *S. chinensis* are 0.119 and −0.106, respectively.

The mt genome of *S. chinensis* encodes all 37 genes typically existing in animal mt genomes, consisting of 13 protein-coding genes (PCG), two ribosomal RNA (rRNA) genes and 22 transfer RNA (tRNA) genes. The mitochondrial gene arrangement of *S. chinensis* is identical to all other lace bugs. Although the mt genome of *S. chinensis* is largest of Tingidae, it is extremely compact. In the mt genome of *S. chinensis*, a total of 31 bp overlaps have been found at 15 gene junctions. The overlaps are ranging from 1 to 8 bp, the longest one locates between *trnW* and *trnC*. However, the mt genome just has 13 bp intergenic sequence without the putative A + T-rich region. The intergenic sequences are at six locations ranging from 1 to 5 bp, the longest one located between *trnP* and *nad6*. The A + T-rich region of this genome is 2215 bp long with the A + T content of 82.58%, and located between the *rrnS* and *trnI* genes. The large length of the A + T-rich region is a principal cause of huge mt genome size.

All of the 22 tRNA genes usually found in insects mitochondrion have been identified in *S. chinensis*, and 14 tRNAs are encoded by the J-strand and the remainder encoded by the N-strand. The gene size of tRNAs ranges from 63 bp (*trnD*, *trnG*, *trnH*, *trnT*, *trnI*, and *trnC*) to 70 bp (*trnV*), and A + T content is ranging from 71.83% (*trnK*) to 91.18% (*trnE*). Like most insect, 21 tRNA genes have typical cloverleaf shaped secondary structure and *trnS_1_* lacks the dihydrouridine (DHU) arm (Wang et al. 2014). The two rRNA genes have been identified on the N-strand in the mt genome: the *rrnL* gene locates between *trnL_1_* and *trnV*, and the *rrnS* gene between the *trnV* and the A + T-rich region. The length of *rrnL* and *rrnS* is 1227 bp and 815 bp, and their A + T content is 80.85% and 82.58%, respectively.

In the tea lace bug mt genome, the total length of all 13 PCGs is 10,984 bp, which is accounting for 65.90% of the total genome. The A + T content of the 13 PCGs ranges from 69.27% (*cox1*) to 84.91% (*atp8*). All of the 13 PCGs initiate translation using ATN codons (ATG for *atp6*, *cox1*, *cox3*, *cob*, *nad1*, and *nad5*; ATT for *atp8*, *nad2*, *nad3*, and *nad4L*; ATA for *cox2*, *nad4*, and *nad6*). Four PCGs (*atp6*, *cox2*, *cox3*, and *nad5*) have incomplete terminal codons consisting of single T nucleotide, and the other PCGs stop with TAA or TAG. The incomplete stop codon T is commonly reported and could produce functional stop codons in polycistronic transcription cleavage and polyadenylation mechanisms (Ojala et al. 1981; Boore 2001). We analysed the amino acid sequences of 13 PCGs with maximum likelihood (ML) method to learn the phylogenetic relationship of *S. chinensis* with other true bugs. In the phylogenetic tree, the three lace bugs were clustered into a branch (Figure 1), it infers that *S. chinensis* is closely related to species of Tingidae. The families Tingidae and Miridae belong to superfamily Miroidea, and Tingidea formed a sister clade to Miridae in the tree.

**Figure 1.**
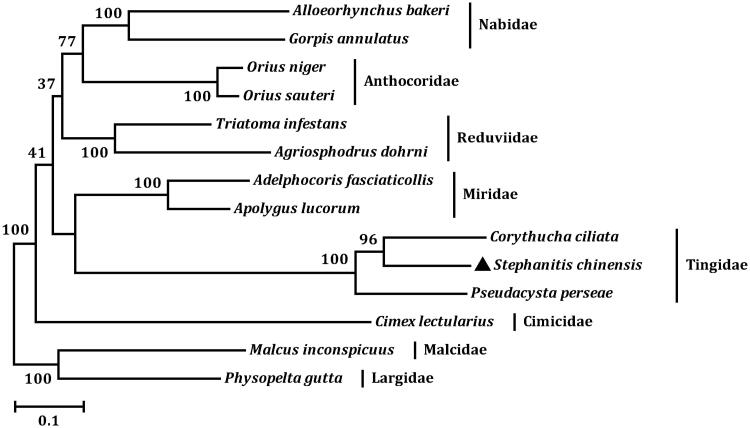
Phylogenetic relationships among six cimicomorphan families inferred from mitochondrial genome sequences. The GenBank accession numbers used for tree construction are as follows: *Corythucha ciliata* (KC756280), *Pseudacysta perseae* (KM278221), *Apolygus lucorum* (HQ902161), *Adelphocoris fasciaticollis* (KJ001714), *Orius sauteri* (KJ671626), *Orius niger* (EU427341), *Cimex lectularius* (KU350871), *Gorpis annulatus* (JF907591), *Alloeorhynchus bakeri* (HM235722), *Agriosphodrus dohrni* (HM071001), *Triatoma infestans* (KY640305), *Physopelta gutta* (EU427343), and *Malcus inconspicuus* (EU427339).
